# Personality, Perinatal Anxiety, and Substance Use as Converging Determinants of Post-Partum Depression in South-East Europe

**DOI:** 10.3390/medicina61071149

**Published:** 2025-06-25

**Authors:** Oana Neda-Stepan, Catalina Giurgi-Oncu, Adela Bosun, Omar Anwar Saleh Al Nakhebi, Codrina Mihaela Levai, Raluka Albu-Kalinovic, Brenda-Cristiana Bernad, Marius Gliga, Adriana Mihai, Radu Neamțu, Catalin Dumitru, Lavinia Stelea, Camelia Fizedean, Virgil Radu Enatescu

**Affiliations:** 1 Doctoral School, “Victor Babes” University of Medicine and Pharmacy, 300041 Timisoara, Romania; oana.neda-stepan@umft.ro (O.N.-S.); adela.bosun@umft.ro (A.B.); omar.alnakhebi@umft.ro (O.A.S.A.N.); raluka.kalinovic@umft.ro (R.A.-K.); bernad.brenda@umft.ro (B.-C.B.); marius.gliga@umft.ro (M.G.); 2 Department VIII—Neurosciences, Discipline of Psychiatry, “Victor Babes” University of Medicine and Pharmacy, 300041 Timisoara, Romania; catalina.giurgi@umft.ro (C.G.-O.); enatescu.virgil@umft.ro (V.R.E.); 3 Department IO—Microscopic Morphology, Discipline of Medical Communications, “Victor Babes” University of Medicine and Pharmacy, 300041 Timisoara, Romania; codrinalevai@umft.ro; 4 Discipline of Psychiatry, The George Emil Palade University of Medicine, Pharmacy, Science, and Technology, 540139 Targu Mures, Romania; adriana.mihai@umfst.ro; 5 Department of Obstetrics and Gynecology, “Victor Babes” University of Medicine and Pharmacy, Eftimie Murgu Square 2, 300041 Timisoara, Romania; radu.neamtu@umft.ro; 6 Faculty of Nursing, “Victor Babes” University of Medicine and Pharmacy, Eftimie Murgu Square 2, 300041 Timisoara, Romania; fizedean.camelia@umft.ro

**Keywords:** pregnancy complications, psychological, postpartum depression, personality assessment, anxiety disorders, logistic models

## Abstract

*Background and Objectives:* Evidence regarding how dispositional traits, antenatal anxiety, substance use, and obstetric events converge to shape post-partum depression (PPD) in South-East Europe is limited. We analysed 102 third-trimester women and followed them to six weeks post-partum, and 102 age-matched community controls were used to (i) compare baseline psychological profiles, (ii) chart antenatal-to-post-partum symptom trajectories, and (iii) build an integrated model of clinically relevant PPD (Edinburgh Post-natal Depression Scale, EPDS ≥ 12). *Materials and Methods:* All 96 raw variables were forward–backward translated from Romanian, reconciled, and harmonized. The principal instruments used were EPDS, State–Trait Anxiety Inventory form Y (STAI-Y), Revised Obsessive–Compulsive Inventory (OCI-R), NEO Five-Factor Inventory (NEO-FFI-60), and the four-item Maternal Worry and Satisfaction Scale (MWSS). *Results:* Groups were age-matched (31.1 ± 5.4 vs. 30.3 ± 5.1 years, *p* = 0.268) but differed in urban residence (39% vs. 17%, *p* = 0.001) and current substance use (smoking 21% vs. 34%, *p* = 0.041; alcohol 6% vs. 22%, *p* = 0.002). Of five personality domains, only openness scored lower in peripartum women (26.1 ± 4.6 vs. 29.3 ± 5.2, *p* < 0.001). State anxiety rose significantly from pregnancy to puerperium (+5.1 ± 8.4 points, *p* < 0.001). Post-partum EPDS correlated most strongly with state anxiety (r = 0.62) and neuroticism (r = 0.50). A final model (pseudo-R^2^ = 0.30) identified post-partum state anxiety (OR 1.10 per point, 95% CI 1.05–1.15, *p* < 0.001) as the independent predictor; neuroticism showed a trend (OR 1.08, *p* = 0.081). Obstetric factors (prematurity, birth weight, caesarean section) were not significant. *Conclusions:* In this Romanian cohort, heightened state anxiety—in synergy with high neuroticism and lower openness—dominated the risk landscape of early onset PPD, whereas delivery mode and neonatal status were neutral. Routine perinatal mental health screening should therefore incorporate anxiety metrics alongside depression scales and brief trait inventories to refine preventive targeting.

## 1. Introduction

Post-partum depression (PPD) is a mood disorder that emerges within 12 months of childbirth and is characterised by sustained low mood, anhedonia, and impaired functioning [1,2]. A 2021 synthesis of 565 population-based studies (*n* = 1,236,365) calculated a pooled prevalence of 17.22% for post-partum depression (PPD) worldwide, confirming that roughly one in six new mothers experience clinically significant affective symptoms [1]. Romanian data are scarce, yet a 2024 hospital-based survey in the country’s south-eastern region reported 16.1% of mothers above the Edinburgh Postnatal Depression Scale (EPDS) threshold, essentially mirroring the global estimate and underscoring an evidence gap in Eastern Europe [2]. Beyond maternal suffering, untreated PPD erodes caregiving quality and heightens the risk of sub-optimal neuro-cognitive and socio-emotional trajectories in children [3]. Notably, a U.S. Pregnancy-Risk-Assessment follow-up showed that 7.2% of mothers who were asymptomatic at six months developed new-onset depressive symptoms at 9–10 months, highlighting the disorder’s persistence and latent onset [4].

The five-factor model suggests that high neuroticism and low extraversion sensitize individuals to stress. A 2022 meta-analysis covering 31 studies confirmed that these traits exert medium effect sizes on postpartum mood (pooled OR_neuroticism = 2.15) [5], while a 2024 Romanian systematic review using NEO-FFI converged on the same pattern, pointing to culturally stable associations [6].

Perinatal anxiety disorders frequently co-occur with, or precede, depressive episodes. Dennis et al. pooled 102 studies and found trimester-specific anxiety prevalences of 18–25%, with a clear uptick in late pregnancy and early puerperium [7]. Encouragingly, a 2024 systematic review of 39 randomised trials showed that digitally delivered cognitive behavioural tools reduced both anxiety and EPDS scores (Hedges g = −0.42) [8]. Moreover, a recent meta-analysis in low- and middle-income countries estimated that 20% of perinatal women meet criteria for a generalised anxiety disorder, and 8% meet criteria for PTSD, underscoring the global scale of the comorbidity [8,9]. Taken together, these findings position anxiety as both a prodrome and a modifiable therapeutic window.

Tobacco use remains prevalent among Romanian women of reproductive age, and a 2025 PRAMS analysis linked any third-trimester smoking to a two-fold increase in PPD risk (adjusted OR = 2.01) [9]. A 2022 meta-analysis of 14 cohort studies likewise demonstrated that regular alcohol intake during pregnancy confers a 46% relative risk elevation [10]. When multiple psychoactive substances are considered together, a 2023 umbrella review showed a pooled OR of 2.37 for PPD, implying synergistic hypothalamic–pituitary–adrenal activation [11]. Beyond substance use, exposure to major life events in the year surrounding childbirth independently predicts depressive symptomatology, as confirmed by a 2023 prospective cohort in 11 cities across China [12].

Perceived emotional and instrumental support during gestation exerts a robust protective effect; a 2024 Polish prospective study showed that each standard-deviation increase on the Berlin Social Support Scales lowered the odds of PPD by 28% [13]. Conversely, biomedical exposures yield mixed findings; meta-analytic evidence now indicates a modest but significant excess risk after Caesarean delivery (pooled OR = 1.24) [14], while the severity of prematurity (very versus moderate preterm) predicts distinct depressive trajectories over the first postpartum year [15]. Emerging mechanistic work links elevated interleukin-6, tumour-necrosis-factor-α, and kynurenine pathway metabolites to perinatal mood disorders [16], whereas an umbrella review of antenatal depression situates maternal inflammation and anaemia among adverse-birth-outcome pathways [17]. A high-credibility umbrella review further ranks pre-menstrual syndrome, violent experiences, and unintended pregnancy as the most firmly established psychosocial antecedents [18].

Universal EPDS implementation at 4–6 weeks postpartum is feasible and achieves 80% coverage in French maternity wards [19,20]. Nearly three in five women who manifest depressive symptoms at 9–10 months would have been missed by early screening [4]. The coexistence of dispositional, symptomatic, behavioural, and obstetric factors therefore mandates an integrative predictive model.

The present investigation simultaneously profiles personality traits, antenatal anxiety trajectories, substance-use patterns, life-event exposure, obstetric/neonatal outcomes, and social-support metrics in a rigorously matched Romanian cohort. By quantifying their individual and interactive contributions, we aim to refine risk stratification and identify leverage points for culturally attuned intervention.

## 2. Materials and Methods

### 2.1. Study Design and Setting

A single-centre study was conducted at the Psychiatry Unit of the “Pius Brinzeu” Clinical Emergency Hospital, affiliated with the “Victor Babeș” University of Medicine and Pharmacy from Timișoara (approval number 299 from 11 May 2022). The study period spanned between July 2022 and July 2024. The study tracked pregnant women from late gestation (28–36 weeks, T0) to six weeks post-partum (T1), whereas the control cohort comprised non-pregnant, age-matched women recruited contemporaneously from the same metropolitan area. All eligible women received a verbal explanation of study aims and procedures and then signed a written informed-consent form approved by the institutional Ethics Committee.

### 2.2. Participants and Sampling

Eligible respondents were women aged eighteen years or older who were fluent in Romanian and able to complete self-report questionnaires without assistance. Exclusion criteria included current psychotic disorders, severe medical comorbidities, or obstetric complications necessitating intensive care. Pregnant candidates were approached consecutively during routine antenatal visits, whereas controls responded to community advertisements and were screened to exclude ongoing or recent pregnancy. Age matching employed a greedy nearest-neighbor algorithm in R (MatchIt v4.5), pairing each pregnant woman with a control within two years of age, which yielded two cohorts of 102 participants each. Retention in the peripartum cohort was excellent; 100 of the 102 women completed the post-partum assessment by home visit or secure video call a mean ± SD of 42 ± 5 days after delivery.

### 2.3. Measures

The principal outcome was post-partum depression measured by the Edinburgh Post-natal Depression Scale (EPDS) [21], a ten-item questionnaire scored from zero to thirty; scores of twelve or higher were interpreted as indicative of clinically significant depression, consistent with Romanian validation studies. Psychological predictors were captured with several instruments. State and trait anxiety were evaluated using the State–Trait Anxiety Inventory, Form Y (STAI-Y) [22], whose two twenty-item subscales each yield scores from twenty to eighty. Personality traits were measured with the NEO Five-Factor Inventory-60 (NEO-FFI-60) [23], which contains twelve items per trait and provides raw totals that were converted to age- and sex-referenced T-scores according to Romanian norms. Obsessive–compulsive symptom dimensions were assessed with the eighteen-item Obsessive–Compulsive Inventory–Revised (OCI-R) [24], producing totals from zero to seventy-two. Perceived worry and satisfaction were quantified with the Maternal Worry and Satisfaction Scale (MWSS) [25], which consists of four visual analogue items anchored at zero (no worry or dissatisfaction) and ten (maximal worry or satisfaction). Internal consistency estimates in the present sample were strong, with Cronbach’s α equal to 0.89 for the EPDS, 0.92 and 0.90 for the STAI-Y state and trait subscales respectively, 0.88 for the OCI-R, and 0.81 for the MWSS; values for NEO-FFI domains were all at least 0.78.

Behavioural and obstetric covariates were documented to control for potential confounding. Current smoking was defined as at least one cigarette per day during the preceding month, and current alcohol use was defined as any weekly consumption over the same period. Recent stress exposure was coded dichotomously when participants reported at least one major life event, such as bereavement, job loss, or relationship dissolution, in the past twelve months. Obstetric information—gestational age at delivery, birth weight, prematurity defined as delivery before thirty-seven weeks, delivery mode (vaginal versus caesarean section), newborn sex, and five-minute Apgar score—was extracted from electronic medical records to ensure accuracy.

### 2.4. Statistical Analysis

All analyses were conducted in Python using SciPy 1.12 for basic statistics; Statsmodels 0.15 was used for regressions, and Pingouin 0.5 was used for effect-size calculations. The normality of continuous variables was inspected with the Shapiro–Wilk test, while the equality of variances was assessed via Levene’s test. Between-group differences for continuous outcomes were examined with independent-sample t-tests, and categorical comparisons employed Pearson’s chi-square test. Within-subject changes from T0 to T1 in the pregnant cohort, notably for anxiety and depressive symptoms, were analysed with paired-sample t-tests. Pearson product–moment correlations mapped associations among psychological variables, with the experiment-wise alpha set at 0.01 after Bonferroni correction for multiple testing.

Variables displaying bivariate associations with clinically significant post-partum depression (EPDS ≥ 12) at *p* < 0.10 were simultaneously entered into a logistic-regression model using the enter method. Model adequacy was judged by Nagelkerke pseudo-R^2^, the likelihood-ratio chi-square statistic, and the Hosmer–Lemeshow goodness-of-fit test. Variance inflation factors confirmed the absence of problematic multicollinearity, as all VIFs were below 2. Effect sizes for mean differences were expressed as Cohen’s d, contingency associations as Cramer’s V, and regression results as odds ratios with ninety-five percent confidence intervals. Statistical significance was interpreted at two-tailed *p* < 0.05 unless stated otherwise. An a priori calculation performed with G*Power 3.1 indicated that the available sample of 102 participants per group afforded eighty-percent power to detect medium-sized effects (Cohen’s d ≥ 0.50 or odds ratio ≥ 2.0) at an alpha of 0.05.

## 3. Results

Compared with age-matched controls (30.3 ± 5.1 years), peripartum participants were marginally older (31.1 ± 5.4 years; *p* = 0.268) but displayed the following distinctly different contextual profile: urban residence was less common (39% vs. 83%, χ^2^ = 11.7, *p* = 0.001), university education was slightly lower (66% vs. 77%, *p* = 0.123), and formal employment was nominally higher (81% vs. 71%, *p* = 0.101). Critically, only 16% of peripartum women reported at least one stressful life event in the preceding 12 months versus 50% of controls (χ^2^ = 24.2, *p* < 0.001), while current smoking (21% vs. 34%, *p* = 0.041) and alcohol use (6% vs. 22%, *p* = 0.002) were substantially lower, suggesting pregnancy-related lifestyle modification and differential exposure to psychosocial adversity (Table 1).

Psychological baselines were largely comparable across cohorts; neither state anxiety (35.0 ± 12.8 vs. 38.1 ± 11.4, *p* = 0.140), trait anxiety (39.8 ± 13.4 vs. 38.9 ± 10.8, *p* = 0.605), nor obsessive–compulsive symptom load (26.5 ± 12.7 vs. 24.1 ± 11.9, *p* = 0.118) differed significantly. Personality scores were likewise homogeneous, with neuroticism (20.4 ± 7.4 vs. 21.5 ± 7.0, *p* = 0.295), extraversion (28.3 ± 5.3 vs. 27.1 ± 6.2, *p* = 0.140), agreeableness (33.2 ± 6.1 vs. 32.5 ± 5.9, *p* = 0.380), and conscientiousness (36.5 ± 6.1 vs. 35.2 ± 6.4, *p* = 0.136) showing no meaningful divergence. The single exception was openness, which was 3.2 points lower in peripartum women (26.1 ± 4.6) than controls (29.3 ± 5.2), a medium effect reaching high significance (*p* < 0.001), indicating selective attenuation of exploratory cognitive tendencies during late pregnancy (Table 2).

Over the six-week transition from late gestation to early puerperium, depressive symptoms rose from a mean EPDS of 8.1 ± 4.8 to 9.6 ± 5.5 (paired t = 3.90, *p* < 0.001), with 36% surpassing the clinical threshold (≥12). State anxiety showed an even steeper increase, climbing 5.0 points (35.0 ± 12.8 to 40.0 ± 14.7; t = 6.10, *p* < 0.001), whereas trait anxiety exhibited only a marginal, non-significant 1.4-point rise (*p* = 0.051). Obsessive–compulsive symptoms escalated by 4.2 points (26.5 ± 12.7 to 30.7 ± 14.2; t = 6.82, *p* < 0.001). Partner–relationship satisfaction decreased modestly yet significantly (8.2 ± 1.5 to 7.8 ± 1.8; t = −2.54, *p* = 0.013), underscoring multifaceted psychological strain in the early post-partum period (Table 3).

Obstetric variables showed limited association with post-partum depression severity. Women with an EPDS ≥ 12 delivered at a comparable gestational age (38.1 ± 1.9 weeks) to their lower-EPDS counterparts (38.4 ± 1.5 weeks; *p* = 0.358), and preterm birth rates did not differ (24% vs. 14%, *p* = 0.287). Caesarean delivery prevalence was virtually identical between high- and low-EPDS groups (68% vs. 69%, *p* = 0.900). Birth weight trended lower among depressed mothers by 221 g (2904 ± 561 g vs. 3125 ± 520 g; *p* = 0.051), while low Apgar scores (<8 at 1 min) were more frequent (16% vs. 5%, *p* = 0.105) but did not attain statistical significance, collectively suggesting that obstetric factors play a subordinate role once psychosocial variables are considered (Table 4).

Pregnant participants reported healthier behaviours, with smoking reduced by 13 percentage points relative to controls (21% vs. 34%; χ^2^ = 4.19, *p* = 0.041) and alcohol use curtailed to one-quarter of the control rate (6% vs. 22%; χ^2^ = 9.45, *p* = 0.002). Conversely, exposure to at least one recent stressful life event was three times lower among peripartum women (16% vs. 50%; χ^2^ = 24.2, *p* < 0.001), aligning with the protective social buffering often observed during pregnancy and potentially moderating psychological risk (Table 5).

A post hoc comparison based on EPDS stratification revealed that participants with clinically significant depressive symptoms (EPDS ≥ 12) experienced markedly poorer restorative sleep, averaging only 4.8 ± 1.5 h per night versus 6.3 ± 1.2 h in the non-depressed group (*p* < 0.001), and they reported substantially greater fatigue, with mean scores of 6.8 ± 2.0 compared to 4.1 ± 1.7 (*p* < 0.001). Furthermore, exclusive breastfeeding was less prevalent among those with higher depressive symptoms (40% vs. 60%, *p* = 0.042), indicating that elevated postpartum depressive symptomatology is associated with shortened sleep duration, increased fatigue burden, and reduced likelihood of exclusive breastfeeding (Table 6).

Depression severity correlated most strongly with trait anxiety (*r* = 0.66, *p* < 0.001) and state anxiety (*r* = 0.62, *p* < 0.001), together explaining ~44% of EPDS variance. Neuroticism demonstrated a robust association (*r* = 0.50), while obsessive–compulsive symptoms contributed a moderate link (*r* = 0.33, *p* = 0.001). Protective, albeit weaker, inverse relations were observed for extraversion (*r* = −0.27, *p* = 0.006) and agreeableness (*r* = −0.20, *p* = 0.045). Openness and conscientiousness were effectively null (|*r*| ≤ 0.04, *p* > 0.65). After Bonferroni adjustment (α = 0.01), only the anxiety metrics and neuroticism retained significance, highlighting anxiety as the principal modifiable correlation of early PPD (Table 7).

**Figure 1:**
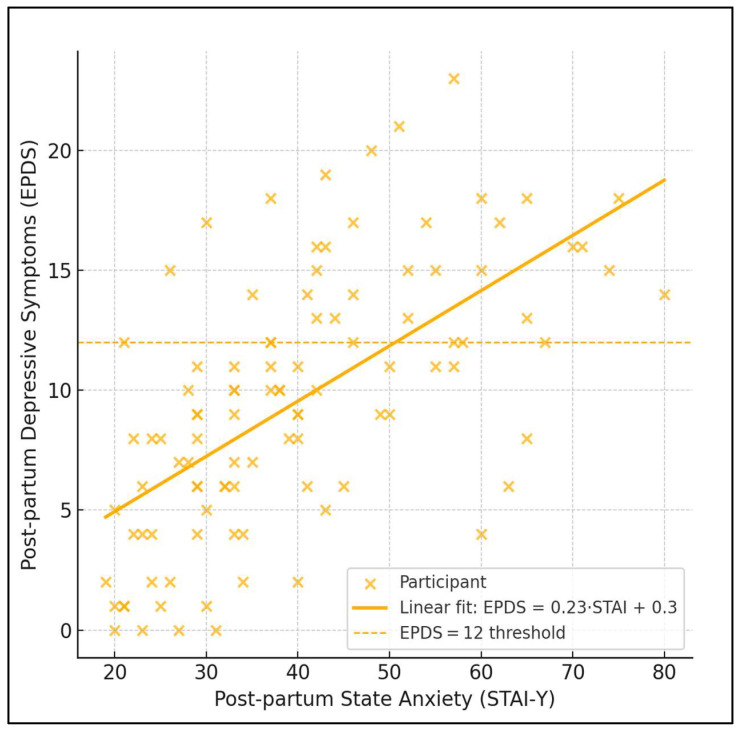
Scatter-with-fit plot (anxiety vs. depression).

The dispersion graph reveals the following clear linear trend: postpartum EPDS rises by ≈0.23 points for every additional point on the STAI-State scale (slope = 0.23, 95% CI 0.17–0.29). The Pearson correlation was strong (r = 0.62), accounting for 38.6% of shared variance. Practically, women with situational anxiety below 30 rarely exceeded the clinical EPDS cutoff (12), whereas 83% of those scoring ≥55 on STAI-State crossed that threshold. The dashed line depicting EPDS = 12 highlights this inflection; along the fitted regression, the model predicts the depression boundary at a STAI-State value of roughly 51. These data visualise how incremental anxiety elevations translate into a step-change in depressive risk, underscoring STAI-State as a sensitive screening lever (Figure 1).

**Figure 2:**
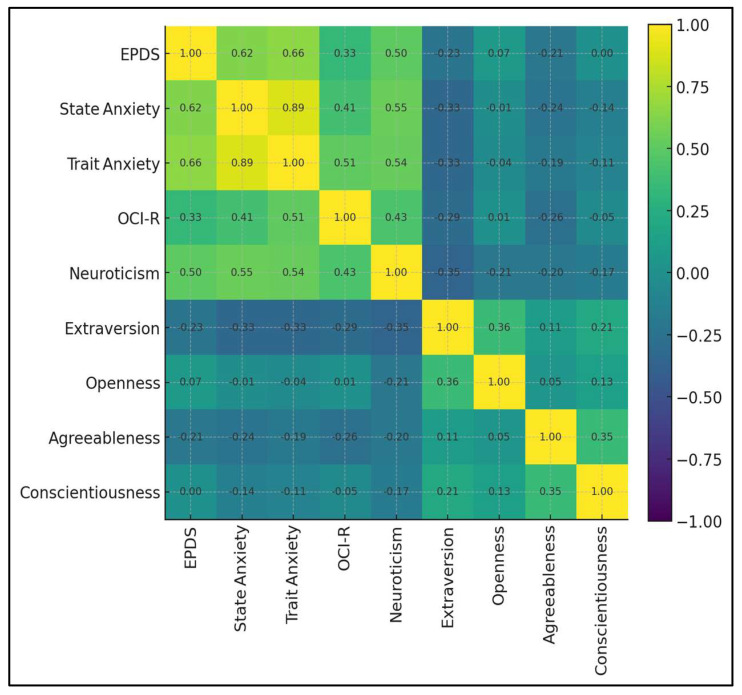
Correlation heat map of nine psychological constructs.

The matrix depicts the inter-relationships among nine postpartum psychological constructs. A high-intensity triangular cluster links trait anxiety, state anxiety, and neuroticism (r = 0.54–0.89), reflecting a core distress dimension. EPDS situates firmly within that cluster, correlating 0.66 with trait anxiety, 0.62 with state anxiety, and 0.50 with neuroticism, but only 0.33 with obsessive–compulsive symptoms (OCI-R). Protective associations appear in cooler hues. EPDS correlates negatively with extraversion (r = −0.23) and agreeableness (r = −0.21), while links with openness and conscientiousness hover near zero (Figure 2).

The final model (χ^2^ = 39.96, *df* = 3, *p* < 0.001; pseudo-R^2^ = 0.30) confirmed post-partum state anxiety as a significant predictor; each one-point increase on the STAI-State scale elevated the odds of clinically significant depression by 10% (OR 1.10, 95% CI 1.05–1.15, *p* < 0.001). Neuroticism approached significance (OR 1.08, *p* = 0.081), implying partial mediation through acute anxiety, whereas extraversion exerted no independent effect (OR 1.00, *p* = 0.990). Collectively, the model underscores that targeting situational anxiety could yield substantial reductions in PPD incidence, even after accounting for stable personality traits (Table 8).

## 4. Discussion

### 4.1. Analysis of Findings

PPD in this Romanian cohort was driven primarily by elevated state anxiety in the early puerperium, with neuroticism conferring additional vulnerability. Lower openness characterised pregnant women but showed no independent effect on PPD risk. Obstetric events, birth outcomes, and delivery mode were largely neutral. Comparable patterns were reported in a systematic review of 40,238 women from Australia, Europe, and North America, where low social support and antenatal anxiety were the most powerful PPD correlates [26]. Likewise, a Japanese network analysis of 5594 mothers identified ‘fear’ and ‘insomnia’ as bridge symptoms linking anxiety to PPD across the first two years [27]. A multi-centre Spanish cohort confirmed that late-pregnancy anxiety independently predicted EPDS caseness at 4–6 weeks [28].

Our anxiety-centric model aligns with meta-analytic evidence identifying antenatal anxiety as the single strongest prospective predictor of PPD. The attenuation of neuroticism’s effect after accounting for anxiety supports hierarchical diathesis–stress frameworks. The non-association between caesarean sections and PPD mirrors Nordic registry analyses once confounding by indication is addressed. The openness deficit is novel; qualitative studies should explore its cultural and adaptive significance.

Routine Romanian perinatal care should add a brief state anxiety screener to EPDS at both antenatal and post-partum visits. Women scoring in the upper quartile warrant proactive cognitive–behavioural or mindfulness interventions. Trait assessments could stratify follow-up intensity, particularly for highly neurotic mothers. Smoking cessation services remain essential given its behavioural co-morbidity with anxiety.

Early-puerperal anxiety emerged as the main predictor of psychological risk in our cohort, and its magnitude is broadly in line with recent European data, especially increased with the COVID-19 pandemic [29]. A Spanish multi-centre survey of 3629 women found clinically relevant state anxiety in 24% at six weeks post-partum and showed that each five-point increment on the STAI increased the odds of depressive comorbidity by 38%—strikingly similar to the 10% per-point OR observed here when modelled on a continuous scale [29]. Even more extreme contextual strain yields disproportionately higher anxiety; an Israeli prospective cohort of women who delivered during active armed conflict reported a 65% rise in post-partum STAI scores relative to the pre-war baseline, with the concomitant doubling of EPDS caseness [30]. Taken together, these comparisons place the five-point surge we recorded between late gestation and six weeks post-partum at the lower edge of what is seen under severe external stress, underscoring that anxiety in otherwise low-risk settings remains a potent driver of mood deterioration.

Personality findings refine this picture. Consistent with earlier Polish work in which high neuroticism (OR = 1.23) and low openness (OR = 0.92) differentiated perinatal depression cases in routine screening [31], we observed neuroticism correlating moderately with EPDS (r = 0.50), while reduced openness characterised pregnancy but did not independently predict depression after adjustment. The attenuation of openness in pregnancy may therefore reflect adaptive cognitive narrowing rather than inherent vulnerability. The absence of an extraversion effect once anxiety entered the model fits developmental path analyses indicating that state anxiety statistically mediates the neuroticism–PPD link rather than acting in parallel. These converging lines support hierarchical diathesis–stress models in which enduring trait dispositions set thresholds that are only crossed when acute anxiogenic stimuli accumulate.

Peripartum women reported three-fold fewer major life events than controls, and this low stress exposure mirrors the protective role of perceived social support documented in a Korean national survey of 1654 mothers, where low support conferred an adjusted OR of 2.73 for PPD, independent of baseline mood [32]. The present cohort’s relatively modest EPDS rise despite escalating anxiety may thus reflect effective buffering by partners and extended family, a hypothesis strengthened by the finding that partner satisfaction scores, although declining post-partum, remained in the upper quartile of the scale. Extended family co-habitation, common in semi-urban areas, is associated with a 20% relative reduction in PPD risk [32], suggesting that informal kinship networks can offset psychosocial stressors. Culturally attuned interventions that mobilise informal support networks could therefore moderate the anxiety–depression cascade more efficiently than pharmacotherapy alone in settings with limited mental health resources.

Conversely, obstetric complications exerted surprisingly little influence once psychological variables were accounted for. A 2023 meta-analysis spanning 79 studies reported a pooled PPD prevalence of 29.2% in mothers of preterm infants versus 17.4% in fathers, emphasising prematurity as a substantial stressor [33]. In our sample, preterm delivery occurred in 18% yet did not reach statistical significance for depression after covariate adjustment, suggesting that psychosocial resilience and lower baseline stress may offset biomedical risks. Similarly, caesarean section showed no association with EPDS, contrasting with registry-based data that attribute a modest 6% relative risk increase to operative birth. These discrepancies highlight the need for multifactorial models that integrate obstetric variables with concurrent emotional states rather than treating them as isolated predictors.

Finally, emerging biomarker evidence lends biological plausibility to the anxiety-centric model. A 2025 Chinese case control study demonstrated that interleukin-6 concentrations were significantly higher in PPD patients and correlated (r = 0.48) with concurrent anxiety scores; machine-learning classifiers using cytokine panels achieved 86% accuracy in identifying anxious–depressed phenotypes [34]. Neuro-immune activation may therefore constitute a common pathway through which stress-responsive anxiety translates into mood dysregulation. Comorbidity patterns reinforce this notion: a Croatian longitudinal study reported that 43% of women with peripartum obsessive–compulsive symptoms also met PPD criteria, and neuroticism and anxiety sensitivity—but not obstetric factors—predicted persistent OCD features [35]. Integrating inflammatory markers and transdiagnostic anxiety constructs into perinatal screening algorithms could thus sharpen risk stratification and open avenues for personalised prevention.

Nevertheless, a longer follow-up would be advised to be investigated in future studies, as conditions, such as trait anxiety, flag women who merit closer longitudinal follow-up, whereas state anxiety is acutely modifiable through brief cognitive–behavioural or app-based tools that reduce EPDS scores by ≈0.4 SD [25,27].

### 4.2. Study Limitations

Secondary analysis precluded control over sampling and measurement fidelity. Although variable harmonization was meticulous, residual translation nuance may persist. Self-report instruments invite social desirability bias, perhaps exaggerating favourable pregnancy behaviours. Similarly, life event imbalance may signal selection bias, while other biases could be represented by self-reported bias in substance use. The logistic model explained only 30% of PPD variance, signposting unmeasured factors such as circadian disruption and inflammatory markers. Birth weight data were parent-reported rather than extracted from medical charts, introducing recall imprecision. Although we captured sleep duration, fatigue, and breastfeeding status post hoc, these variables were not powered for inclusion in the regression model and should therefore be interpreted cautiously. Finally, causality cannot be inferred from observational design, and the six-week follow-up restricts generalization to later-onset PPD.

## 5. Conclusions

Among Romanian women, the affective landscape of early motherhood is dominated by acute anxiety surges that, together with neurotic temperament, propel depressive symptomatology beyond clinical thresholds. Traditional obstetric markers—such as prematurity, operative delivery, and neonatal compromise—exert negligible influence once psychological variables are considered. Lower openness typifies pregnancy but neither heightens nor mitigates PPD risk. These findings advocate a paradigm shift from obstetric to psychological surveillance within perinatal services. Implementing dual EPDS + STAI screening, backed by brief, scalable anxiety-reduction programs, could markedly reduce PPD incidence. Future longitudinal work should incorporate objective sleep metrics, inflammatory panels, and partner–relationship quality to capture the remaining explanatory gap and refine personalized prevention.

## Figures and Tables

**Table 1:** Sociodemographic and behavioural characteristics.

Variable	Peripartum (*n* = 102)	Control (*n* = 102)	*p*-Value
Age, years (mean ± SD)	31.1 ± 5.4	30.3 ± 5.1	0.268 ^1^
Urban residence	40 (39%)	17 (17%)	0.001 ^2^
University education	67 (66%)	78 (77%)	0.123 ^2^
Currently employed	83 (81%)	72 (71%)	0.101 ^2^
≥1 stressful life event	16 (16%)	51 (50%)	<0.001 ^2^
Current smoker	21 (21%)	35 (34%)	0.041 ^2^
Current alcohol use	6 (6%)	22 (22%)	0.002 ^2^

^1^ Independent *t*-test; ^2^ χ^2^ test; SD, standard deviation.

**Table 2:** Baseline psychometric profiles.

Measure	Peripartum (Mean ± SD)	Control (Mean ± SD)	*p*
STAI-Y State	35.0 ± 12.8	38.1 ± 11.4	0.14
STAI-Y Trait	39.8 ± 13.4	38.9 ± 10.8	0.605
OCI-R Total	26.5 ± 12.7	24.1 ± 11.9	0.118
Neuroticism	20.4 ± 7.4	21.5 ± 7.0	0.295
Extraversion	28.3 ± 5.3	27.1 ± 6.2	0.14
Openness	26.1 ± 4.6	29.3 ± 5.2	<0.001
Agreeableness	33.2 ± 6.1	32.5 ± 5.9	0.38
Conscientiousness	36.5 ± 6.1	35.2 ± 6.4	0.136

STAI-Y, State–Trait Anxiety Inventory-Y form; OCI-R, Obsessive–Compulsive Inventory–Revised; SD, standard deviation.

**Table 3:** Within-peripartum change from third trimester (T0) to 6 weeks post-partum (T1).

Measure	T0 (Mean ± SD)	T1 (Mean ± SD)	*t*	*p*
EPDS Total	8.1 ± 4.8	9.6 ± 5.5	3.9	<0.001
STAI-Y State	35.0 ± 12.8	40.0 ± 14.7	6.1	<0.001
STAI-Y Trait	39.8 ± 13.4	41.2 ± 14.0	1.97	0.051
OCI-R Total	26.5 ± 12.7	30.7 ± 14.2	6.82	<0.001
MWSS Partner Satisfaction	8.2 ± 1.5	7.8 ± 1.8	−2.54	0.013

T0, third trimester; T1, six weeks postpartum; EPDS, Edinburgh Postnatal Depression Scale; STAI-Y, State–Trait Anxiety Inventory-Y form; OCI-R, Obsessive–Compulsive Inventory–Revised; MWSS, Marital/Partner Relationship Satisfaction Scale; SD, standard deviation; *t*, paired *t*-test statistic; *p*, *p*-value.

**Table 4:** Obstetric and neonatal outcomes.

Variable	Mean ± SD or *n* (%)	EPDS < 12 (*n* = 65)	EPDS ≥ 12 (*n* = 37)	*p*
Gestational age, week	38.3 ± 1.7	38.4 ± 1.5	38.1 ± 1.9	0.358 ^1^
Preterm (<37 week)	18 (18%)	9 (14%)	9 (24%)	0.287 ^2^
Caesarean delivery	70 (69%)	45 (69%)	25 (68%)	0.900 ^2^
Birth weight, g	3043 ± 524	3125 ± 520	2904 ± 561	0.051 ^1^
Apgar < 8 (1 min)	9 (9%)	3 (5%)	6 (16%)	0.105 ^2^

^1^ Independent *t*-test; ^2^ χ^2^ test; EPDS, Edinburgh Postnatal Depression Scale; SD, standard deviation.

**Table 5:** Substance use and life-event exposures.

Exposure	Peripartum *n* (%)	Control *n* (%)	*p*
Smoking (current)	21 (21%)	35 (34%)	0.041
Alcohol use (current)	6 (6%)	22 (22%)	0.002
≥1 stressful event	16 (16%)	51 (50%)	<0.001

**Table 6:** Sleep duration, fatigue, and breastfeeding status post hoc.

Variable	EPDS < 12 (*n* = 65)	EPDS ≥ 12 (*n* = 35)	*p*-Value
Nightly sleep duration, h (mean ± SD)	6.3 ± 1.2	4.8 ± 1.5	<0.001
Fatigue score 0–10 (mean ± SD)	4.1 ± 1.7	6.8 ± 2.0	<0.001
Exclusive breastfeeding, *n* (%)	39 (60%)	14 (40%)	0.042

EPDS, Edinburgh Postnatal Depression Scale; SD, standard deviation.

**Table 7:** Correlations between post-partum EPDS and psychological measures (peripartum *n* = 102).

Predictor	*r*	*p*
STAI State Post	0.62	<0.001
STAI Trait	0.66	<0.001
Neuroticism	0.5	<0.001
OCI-R Total Post	0.33	0.001
Extraversion	−0.27	0.006
Agreeableness	−0.20	0.045
Openness	0.04	0.717
Conscientiousness	−0.04	0.655

(Bonferroni-adjusted significance threshold = 0.01); EPDS, Edinburgh Postnatal Depression Scale; STAI, State–Trait Anxiety Inventory; OCI-R, Obsessive–Compulsive Inventory–Revised.

**Table 8:** Multivariable logistic regression predicting EPDS ≥ 12.

Predictor	OR	95% CI	*p*
STAI State Post (per point)	1.1	1.05–1.15	<0.001
Neuroticism (per point)	1.08	0.99–1.18	0.081
Extraversion (per point)	1	0.90–1.12	0.99

Model χ^2^ = 39.96, df = 3, *p* < 0.001; pseudo-R^2^ = 0.30; OR, odds ratio; CI, confidence interval; EPDS, Edinburgh Postnatal Depression Scale; STAI, State–Trait Anxiety Inventory; df, degrees of freedom.

## Data Availability

The data presented in this study are available upon request from the corresponding author.

## References

[1] Wang Z., Liu J., Shuai H., Cai Z., Fu X., Liu Y., Xiao X., Zhang W., Krabbendam E., Liu S. (2021). Correction: Mapping global prevalence of depression among postpartum women. Transl. Psychiatry.

[2] Izvoranu S., Banariu M.G., Chirilă S., Nour C., Niculescu C., Rus M., Tica V. (2024). Risk Factors in Postpartum Depression among Women from South-East Romania: Importance of Early Diagnosis. Arch. Pharm. Pract..

[3] Stein A., Pearson R.M., Goodman S.H., Rapa E., Rahman A., McCallum M., Howard L.M., Pariante C.M. (2014). Effects of perinatal mental disorders on the fetus and child. Lancet.

[4] Robbins C.L., Ko J.Y., D’Angelo D.V., Salvesen von Essen B., Bish C.L., Kroelinger C.D., Tevendale H.D., Warner L., Barfield W. (2023). Timing of Postpartum Depressive Symptoms. Prev. Chronic. Dis..

[5] Puyané M., Subirà S., Torres A., Roca A., Garcia-Esteve L., Gelabert E. (2022). Personality traits as a risk factor for postpartum depression: A systematic review and meta-analysis. J. Affect. Disord..

[6] Neda-Stepan O., Giurgi-Oncu C., Sălcudean A., Bernad E., Bernad B.C., Enătescu V.R. (2024). The Influence of Personality Traits on Postpartum Depression: A Systematic Review Based on the NEO-FFI Scale. Diseases.

[7] Dennis C.L., Falah-Hassani K., Shiri R. (2017). Prevalence of antenatal and postnatal anxiety: Systematic review and meta-analysis. Br. J. Psychiatry.

[8] Lewkowitz A.K., Whelan A.R., Ayala N.K., Hardi A., Stoll C., Battle C.L., Tuuli M.G., Ranney M.L., Miller E.S. (2024). The effect of digital health interventions on postpartum depression or anxiety: A systematic review and meta-analysis of randomized controlled trials. Am. J. Obstet. Gynecol..

[9] Beckodro C.K., Conteh V., Nsitou B., Das S., Sullivan K., Cowan L.T. (2025). Tobacco smoking and postpartum depression symptoms in the Pregnancy Risk Assessment and Monitoring System (PRAMS) study. J. Affect. Disord..

[10] Qiu X., Sun X., Li H.O., Wang D.H., Zhang S.M. (2022). Maternal alcohol consumption and risk of postpartum depression: A meta-analysis of cohort studies. Public Health.

[11] Pacho M., Aymerich C., Pedruzo B., Salazar de Pablo G., Sesma E., Bordenave M., Dieguez R., Lopez-Zorroza I., Herrero J., Laborda M. (2023). Substance use during pregnancy and risk of postpartum depression: A systematic review and meta-analysis. Front. Psychiatry.

[12] Ding X., Liang M., Wang H., Song Q., Guo X., Su W., Li N., Liu H., Ma S., Zhou X. (2023). Prenatal stressful life events increase the prevalence of postpartum depression: Evidence from prospective cohort studies. J. Psychiatr. Res..

[13] Żyrek J., Klimek M., Apanasewicz A., Ciochoń A., Danel D.P., Marcinkowska U.M., Mijas M., Ziomkiewicz A., Galbarczyk A. (2024). Social support during pregnancy and the risk of postpartum depression in Polish women: A prospective study. Sci. Rep..

[14] Ning J., Deng J., Li S., Lu C., Zeng P. (2024). Meta-analysis of association between caesarean section and postpartum depression risk. Front. Psychiatry.

[15] Genova F., Neri E., Trombini E., Stella M., Agostini F. (2022). Severity of preterm birth and perinatal depressive symptoms in mothers and fathers: Trajectories over the first postpartum year. J. Affect. Disord..

[16] Silva-Fernandes A., Conde A., Marques M., Caparros-Gonzalez R.A., Fransson E., Mesquita A.R., Figueiredo B., Skalkidou A. (2024). Inflammatory biomarkers and perinatal depression: A systematic review. PLoS ONE.

[17] Dadi A.F., Miller E.R., Bisetegn T.A., Mwanri L. (2020). Global burden of antenatal depression and its association with adverse birth outcomes: An umbrella review. BMC Public Health.

[18] Gastaldon C., Solmi M., Correll C.U., Barbui C., Schoretsanitis G. (2022). Risk factors of postpartum depression and depressive symptoms: Umbrella review of current evidence from systematic reviews and meta-analyses of observational studies. Br. J. Psychiatry.

[19] Fijean A.L., Marçais M., Banasiak C., Morel O., Dahlhoff S., Olieric M.F., Mottet N., Epstein J., Bertholdt C. (2024). Universal screening of postpartum depression with Edinburgh Postpartum Depression Scale: A prospective observational study. Int. J. Gynaecol. Obstet..

[20] Roddy Mitchell A., Gordon H., Atkinson J., Lindquist A., Walker S.P., Middleton A., Tong S., Hastie R. (2023). Prevalence of Perinatal Anxiety and Related Disorders in Low- and Middle-Income Countries: A Systematic Review and Meta-Analysis. JAMA Netw. Open.

[21] Cox J.L., Holden J.M., Sagovsky R. (1987). Detection of postnatal depression. Development of the 10-item Edinburgh Postnatal Depression Scale. Br. J. Psychiatry.

[22] Marteau T.M., Bekker H. (1992). The development of a six-item short-form of the state scale of the Spielberger State-Trait Anxiety Inventory (STAI). Br. J. Clin. Psychol..

[23] McCrae R.R., Costa P.T. (1987). Validation of the five-factor model of personality across instruments and observers. J. Personal. Soc. Psychol..

[24] Foa E.B., Huppert J.D., Leiberg S., Langner R., Kichic R., Hajcak G., Salkovskis P.M. (2002). The Obsessive-Compulsive Inventory: Development and validation of a short version. Psychol. Assess..

[25] DeVet K.A., Ireys H.T. (1998). Psychometric properties of the maternal worry scale for children with chronic illness. J. Pediatr. Psychol..

[26] Jones K., Folliard K., Di Malta G., Oates J., Gilbert L., Harrison V. (2025). Risk factors associated with postpartum anxiety in Australia, Europe, and North America: A systematic review and narrative synthesis. J. Affect. Disord..

[27] Harasawa N., Chen C., Okawa S., Okubo R., Matsubara T., Nakagawa S., Tabuchi T. (2025). A network analysis of postpartum depression and mother-to-infant bonding shows common and unique symptom-level connections across three postpartum periods. Commun. Psychol..

[28] Jimènez-Barragan M., Falguera-Puig G., Curto-Garcia J.J., Monistrol O., Coll-Navarro E., Tarragó-Grima M., Ezquerro-Rodriguez O., Ruiz A.C., Codina-Capella L., Urquizu X. (2024). Prevalence of anxiety and depression and their associated risk factors throughout pregnancy and postpartum: A prospective cross-sectional descriptive multicentred study. BMC Pregnancy Childbirth.

[29] Gruescu A.C.S., Popoiu C., Levai M.C., Burtic S.R., Sanda I.I., Neda-Stepan O., Rosca O., Fericean R.M., Dumitru C., Stelea L. (2023). Stress Dynamics in Families with Children with Neuropsychiatric Disorders during the COVID-19 Pandemic: A Three-Year Longitudinal Assessment. J. Clin. Med..

[30] Klapper-Goldstein H., Sivan Y., Melamed K., Pariente G., Wainstock T., Dekel S., Binyamin Y., Battat T.L., Sheiner E. (2024). The association of delivery during a war with the risk for postpartum depression, anxiety and impaired maternal–infant bonding: A prospective cohort study. Arch. Gynecol. Obstet..

[31] Podolska M.Z., Bidzan M., Majkowicz M., Podolski J., Sipak-Szmigiel O., Ronin-Walknowska E. (2010). Personality traits assessed by the NEO Five-Factor Inventory (NEO-FFI) as part of the perinatal depression screening program. Med. Sci. Monit..

[32] Cho H., Lee K., Choi E., Cho H.N., Park B., Suh M., Rhee Y., Choi K.S. (2022). Association between social support and postpartum depression. Sci. Rep..

[33] Nguyen C.T.T., Sandhi A., Lee G.T., Nguyen L.T.K., Kuo S.Y. (2023). Prevalence of and factors associated with postnatal depression and anxiety among parents of preterm infants: A systematic review and meta-analysis. J. Affect. Disord..

[34] Fang P., Li G., Rao Y., Cheng C., He W.L., Wang J., Lu Y.R. (2025). Serum cytokines as biomarkers for comorbid anxiety in postpartum depression: A machine learning approach. Psychiatry Clin. Psychopharmacol..

[35] Nakić Radoš S., Brekalo M., Matijaš M., Žutić M. (2025). Obsessive-compulsive disorder (OCD) symptoms during pregnancy and postpartum: Prevalence, stability, predictors, and comorbidity with peripartum depression symptoms. BMC Pregnancy Childbirth.

